# Androgen conversion in osteoarthritis and rheumatoid arthritis synoviocytes – androstenedione and testosterone inhibit estrogen formation and favor production of more potent 5α-reduced androgens

**DOI:** 10.1186/ar1769

**Published:** 2005-06-10

**Authors:** Martin Schmidt, Claudia Weidler, Heidrun Naumann, Sven Anders, Jürgen Schölmerich, Rainer H Straub

**Affiliations:** 1 Institute of Biochemistry II, Hospital of the Friedrich-Schiller-University, Jena, Germany; 2 Department of Internal Medicine I, University Hospital Regensburg, Regensburg, Germany; 3 Department of Orthopedic Surgery, University Regensburg, Bavarian Red Cross Hospital, Bad Abbach, Germany

## Abstract

In synovial cells of patients with osteoarthritis (OA) and rheumatoid arthritis (RA), conversion products of major anti-inflammatory androgens are as yet unknown but may be proinflammatory. Therefore, therapy with androgens in RA could be a problem. This study was carried out in order to compare conversion products of androgens in RA and OA synoviocytes. In 26 OA and 24 RA patients, androgen conversion in synovial cells was investigated using radiolabeled substrates and analysis by thin-layer chromatography and HPLC. Aromatase expression was studied by immunohistochemistry. Dehydroepiandrosterone (DHEA) was converted into androstenediol, androstenedione (ASD), 16αOH-DHEA, 7αOH-DHEA, testosterone, estrone (E1), estradiol (E2), estriol (E3), and 16αOH-testosterone (similar in OA and RA). Surprisingly, levels of E2, E3, and 16α-hydroxylated steroids were as high as levels of testosterone. In RA and OA, 5α-dihydrotestosterone increased conversion of DHEA into testosterone but not into estrogens. The second androgen, ASD, was converted into 5α-dihydro-ASD, testosterone, and negligible amounts of E1, E2, E3, or 16αOH-testosterone. 5α-dihydro-ASD levels were higher in RA than OA. The third androgen, testosterone, was converted into ASD, 5α-dihydro-ASD, 5α-dihydrotestosterone, and negligible quantities of E1 and E2. 5α-dihydrotestosterone was higher in RA than OA. ASD and testosterone nearly completely blocked aromatization of androgens. In addition, density of aromatase-positive cells and concentration of released E2, E3, and free testosterone from superfused synovial tissue was similar in RA and OA but estrogens were markedly higher than free testosterone. In conclusion, ASD and testosterone might be favorable anti-inflammatory compounds because they decrease aromatization and increase anti-inflammatory 5α-reduced androgens. In contrast, DHEA did not block aromatization but yielded high levels of estrogens and proproliferative 16α-hydroxylated steroids. Androgens were differentially converted to pro- and anti-inflammatory steroid hormones via diverse pathways.

## Introduction

Adrenal and gonadal androgens such as dehydroepiandrosterone (DHEA), androstenedione (ASD), and testosterone have anti-inflammatory properties mediated by blocking the secretion of interleukin (IL)-1β, IL-6, tumor necrosis factor (TNF), and other proinflammatory mediators [1-7]. The more potent, pure androgen 5α-dihydrotestosterone has been found to repress the NFκB-mediated activation of the human IL-6 gene promoter in human fibroblasts [8], and it also inhibits T cell proliferation [9]. An open, double-blind therapy study with testosterone demonstrated remarkable benefits in patients with RA [10,11]. As a prerequisite for further therapeutic administration of androgens to patients with RA, it is important to know how androgens can be converted into downstream hormones in affected synovial tissue.

**Figure 1:**
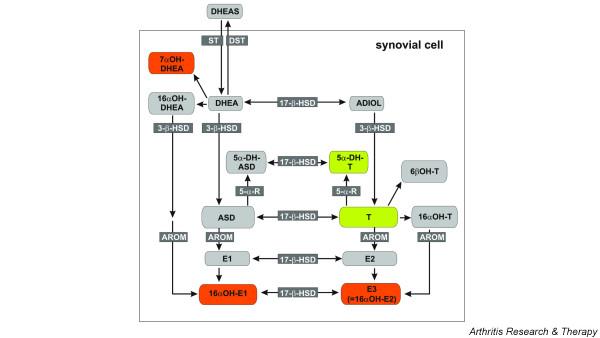
Complexity of androgen conversion in peripheral cells. DHEAS, DHEA, and ASD are the major androgen precursors, which are released from the adrenal gland (particularly relevant in postmenopausal women). These androgens enter the peripheral cell to be converted to downstream metabolites using diverse enzyme pathways. 3β-HSD, 3β-hydroxysteroid dehydrogenase (converts delta 5 androgens into delta 4 androgens); 5α-DH, 5α-dihydro; 5-α-R, 5α-reductase; 17β-HSD, 17β-hydroxysteroid dehydrogenase; ADIOL, androstenediol; ASD, androstenedione; AROM, aromatase; DHEA, dehydroepiandrosterone; DHEAS, DHEA sulfate; DST, DHEA sulfotransferase; E1, estrone; E2, 17β-estradiol; E3, 16α-hydroxylated E2 (also known as estriol); OH, hydroxyl group at the indicated position; ST, sulfatase; T, testosterone.

Apart from gonadal cells, different peripheral cells are able to convert androgens into downstream steroid hormone products such as estrogens [12-16]. Figure 1 demonstrates the complexity of intracellular steroid hormone conversion (intracrinology). In a recent preliminary study in mixed synovial cells of three patients with rheumatoid arthritis (RA) and osteoarthritis (OA), we demonstrated that DHEA can be converted to testosterone, estrone (E1), and 17β-estradiol (E2) [17]. In collagen type II arthritic animals, others have demonstrated that DHEA can be converted into the proinflammatory steroid hormone 7αOH-DHEA, due to increased expression of the P450 enzyme CYP7B [18]. This has been confirmed in RA synovial fibroblasts (J Dulos, personal communication). However, unlike in the case of DHEA, it is presently unknown how ASD and testosterone can be converted into downstream hormones in mixed synovial cells of patients with RA, and whether this conversion is different in OA patients. This is important to know because the delta 4 androgens ASD and testosterone are more potent and, thus, may be used in clinical trials in patients with RA [10,11]. If ASD and testosterone are also converted into more proinflammatory downstream steroid hormones their therapeutic administration may be a problem.

This study was initiated in order to investigate conversion of DHEA, ASD, and testosterone in synovial tissue of patients with RA and OA. We functionally tested hormone conversion in mixed synovial cells of RA and OA patients and tried to find the factors that influence these particular enzyme steps in primary synovial cells. We used mixed primary synovial cells in order to give an *in vivo *figure of steroid conversion. Furthermore, we studied aromatase expression in synovial tissues of patients with RA and OA.

## Patients and methods

### Patients

In this study, 25 patients with long-standing RA who fulfilled the American College of Rheumatology criteria for RA [19] and 26 patients with OA were included. These patients underwent elective knee joint replacement surgery, which is typically carried out in the late chronic phase of the disease. All our investigations are thus related to long-standing chronic disease in an advanced phase. Patients were informed about the purpose of the study and gave written consent. The study was approved by the Ethics Committee of the University of Regensburg, Germany. Basic clinical and laboratory data are given in Table 1. Parameters such as erythrocyte sedimentation rate, C-reactive protein, and rheumatoid factor were measured by standard techniques as previously described [20].

### Synovial tissue preparation

Synovial tissue samples were obtained immediately after opening the knee joint capsule, preparation of which was previously described [21]. A piece of synovial tissue of up to 9 cm^2 ^was dissected. Six pieces of about 16 mm^2 ^were loaded into separate superfusion chambers (see below), a larger piece of the same tissue was used to isolate primary mixed synovial cells (see below), and approximately eight pieces of the same synovial area were used for histology: samples intended for hematoxylin-eosin (HE) staining and alkaline phosphatase-anti-alkaline phosphatase (APAAP) staining were immediately placed in protective freezing medium (Tissue Tek; Sakura Finetek, Zoeterwoude, The Netherlands) and then quick frozen. Tissue samples for the detection of aromatase were fixed for 12 to 24 hr in phosphate buffered saline (PBS) containing 3.7% formaldehyde and then incubated in PBS with 20% sucrose for 12 to 24 hr. Thereafter, tissue was embedded in Tissue Tek and quick frozen. All tissue samples were stored at -80°C.

### Histological evaluation and determination of density of aromatase-positive cells

Histological evaluation was carried out as previously described [20]. Using 5 to 7 μm thick sections, cell density and lining layer thickness were determined for about 45 sections from at least three different tissue samples per patient (HE stain). The overall cell density was determined by counting all stained cell nuclei in 17 randomly selected high-power fields of view (400×). The lining layer thickness was analyzed by averaging the number of cells in a lining layer cross section at nine different locations (400×). To determine the number of T cells (CD3; Dako, Hamburg, Germany), macrophages (CD163; Dako), and vessels (collagen IV; Dako), eight cryosections were investigated using APAAP staining and then the number of identified structures was averaged from 17 randomly selected high-power fields (400×). The number of investigated high-power fields was derived from a pioneering histological study by Bresnihan *et al*. [22].

For the determination of the density of synovial aromatase-positive cells, six to eight cryosections (5 to 7 μm thick) were used for immunohistochemistry with a monoclonal primary antibody against aromatase (Serotec GmbH, Düsseldorf, Germany), and an alkaline phosphatase-conjugated secondary antibody (Dako Cytomation). Staining of the labeled cells was achieved by the substrate BCIP/NBT. The numbers of aromatase-positive cells per mm^2 ^were determined by averaging the number of stained cells in 17 randomly selected high-power fields of view (400×).

### Isolation and culture of primary mixed synovial cells

Mixed synovial cells were isolated by enzymatic digestion of synovial tissue for 1 to 2 hr at 37°C using Dispase (Grade II; Boehringer, Mannheim, Germany). The synovial cells were re-suspended in RPMI 1640 medium (Sigma, Taufkirchen, Germany), supplemented with 10% fetal calf serum (FCS), 1% penicillin/streptomycin, 0.1% amphotericin B, and 4 ml/l ciprofloxacin. The cells were stored for 24 hr in teflon bags (Heraeus, Hanau, Germany) for immediate 4°C express shipping to the University of Jena (to MS and HN). After removal from the teflon bags, synoviocytes were washed twice with serum-free RPMI medium (Biochrom, Berlin, Germany). Roughly, 3 × 10^5^ to 4 × 10^5^ viable cells per well were placed into six-well plates in a volume of 3 ml and incubated for 3 hr. There was no difference in viability between cells obtained from OA and RA patients. During culture, cells were kept in a humidified atmosphere with 5% CO2 at a temperature of 37°C. After 3 hr, cells were subjected to incubation with radiolabeled DHEA, ASD, or testosterone with/without additional test compounds (see below).

The percentage of different types of synoviocytes was tested by specific antibodies against prolyl 4 hydroxylase (for the synoviocyte type B = fibroblasts; Calbiochem, Bad Soden, Germany) and CD163 (synoviocyte type A = macrophages; Dako). In preliminary experiments with primary early culture mixed synoviocytes from three patients with RA and three patients with OA, we detected that 26 ± 3% of cells were positive for CD163 (i.e. macrophages) and 37 ± 3% were positive for prolyl 4 hydroxylase (i.e. fibroblasts). There was a significant difference between RA and OA patients with respect to percentage of CD163 positive cells (36 ± 3 vs 15 ± 3%, *p *< 0.001), which was not observed for prolyl 4 hydroxylase positive cells (37 ± 4 vs 38 ± 4%, NS). This suggests that the results with primary early culture mixed synoviocytes from RA patients were more influenced by macrophages (CD 163) as compared with cultures from OA patients.

### Incubation with radiolabeled steroids and steroid extraction

Solvents and other reagents were purchased from Merck (Darmstadt, Germany), if not stated otherwise. Unlabeled steroids were from Sigma and from Steraloids (Newport, RI, USA). Stock solutions were prepared in ethanol. Estrogen stocks contained 2.5mM ascorbic acid. Radiolabeled steroids were purchased from PerkinElmer (Rodgau, Germany). Three hours after plating, the radiolabeled substrates were added to the cells for another 48 hr at a final concentration of 250 nM. Substrates used were [4-^14^C]androstenedione (ASD, 4-androsten-3,17-dione, 1983.2 MBq/mmol), [4-^14^C]dehydroepiandrosterone (DHEA, 5-androsten-3β-ol-17-one, 2053 MBq/mmol), or [4-^14^C]testosterone (testosterone, 4-androsten-17β-ol-3-one, 1983.2 MBq/mmol). The time was chosen because it was well within the time window of linear product accumulation for the metabolites as analyzed by thin-layer chromatography (TLC) (data not shown). In one set of experiments, cells were treated with the non-aromatizable androgen 5α-dihydrotestosterone (100 nM) in parallel with radiolabeled substrates. After incubation, culture supernatants were transferred to polypropylene tubes and centrifuged at 4°C at 600 × *g *for 5 min. Steroids were extracted twice with 3ml cold ethyl acetate. The exact concentration of radiolabeled steroid applied to each well and the extraction efficiencies were monitored by liquid scintillation counting of aliquots: recoveries of radioactivity in the organic phase varied insignificantly for the different substrates and were on average more than 98% for ASD, 96% for DHEA, and 99% for testosterone. The stable extracts were lyophilized in a speed-vac concentrator (Saur, Reutlingen, Germany) and stored at -20°C until analysis.

### Two-dimensional TLC of steroids

The separation of steroids was done as previously described [14], with minor modifications as given below. Lyophilized extracts were dissolved in 50 μl ethanol, spotted on silica gel 60 F254 TLC aluminum sheets (Merck) together with a mixture of unlabeled carrier steroids. These mixtures routinely contained DHEA, androstenediol, ASD, testosterone, E1, E2, and 16aOH-E2 (E3, Estriol). Additional steroids were included as necessary to verify co-migration of other metabolites with their respective standards. The first separation was done in toluene:methanol (90:10). After drying, the second development was done in chloroform:diethylether (50:50). For identification of spots, the TLC plates were stained with copper acetate in phosphoric acid as previously described [14]. Radioactivity on the TLC plates was quantified by radioimaging (FLA 3000; Fuji-Raytest, Straubenhardt, Germany). Spots were assigned only if their intensity was more than two standard deviations above background. All TLC analyses were repeated twice for each sample. The results were calculated and given as pmoles of steroid produced by 10^6 ^cells in a 48-hr incubation period.

### HPLC of steroids

To verify the identity of several metabolites, reverse phase HPLC (RP-HPLC) was used. Samples were separated by TLC as described above, but without staining of standard compounds. The areas of interest were identified by radioimaging, excised from the TLC sheets and extracted twice with 700 μl ethyl acetate. The combined extracts were dried in a speed-vac concentrator. The samples were dissolved in 12 μl ethanol containing the appropriate mixture of reference steroids. Analyses were carried out on a radio-HPLC system consisting of an online degasser DGU-14A, a gradient former FCV-10ALVP, a LC-10ATVP pump, a SPD-10AVP UV-detector (all from Shimadzu, Duisburg, Germany), a Rheodyne 7725i injection valve and a flow scintillation detector 505TR (PerkinElmer) equipped with a 500 μl homogenous flow cell. A 3-ml quantity of liquid scintillation cocktail (Ultima Flo-M; PerkinElmer) per ml solvent was mixed online. Alternatively, for analyses where very low amounts of radioactivity were expected, fractions were collected into vials where they were mixed with liquid scintillation cocktail, and counted off-line in a standard scintillation detector.

Separations were done on LiChrospher 100 RP-18e (5 μm) columns (250 × 4 mm) (Merck) immersed in a water-bath kept at 35°C. Flow rates were 1 ml/min. Two solvent systems were used, depending on the hydrophobicity of the analytes of interest: system 1 consisted of methanol:water (50:50) and system 2 was methanol:water (65:35). System 1 was used for identification of 5α-reduced androgens. Retention times of standards were ASD 8.1 min, testosterone 9.9 min, DHEA 11.9 min, 5α-dihydro-ASD 12.9 min, and 5α-dihydrotestosterone 15.8 min; the minimum resolution was 2.0. System 2 was used for complete separation and identification of the most hydrophilic metabolites, which could not be completely resolved in the two-dimensional TLC system. Retention times of these steroids were E3 7.5 min, 6βOH-testosterone 9.3 min, 16αOH-androstenediol 11.0 min, 16αOH-testosterone 12.2 min, and 7αOH-DHEA 14.2 min; the minimum resolution was 2.0.

### Superfusion of synovial tissue and determination of superfusate steroids

This technique has been recently described [20]. Six pieces of synovial tissue sample were placed in superfusion chambers and then superfused with serum-free culture medium (RPMI 1640; Sigma) for 2 hr at 37°C using a flow rate of 66 μl/min. Superfusate was collected after 2 hr and stored at -30°C for later bulk analysis of E2, E3, and free testosterone by ELISA (IBL, Hamburg, Germany). Detection limits for E2, E3, and free testosterone: 59, 70, and 0.5 pmol /l, respectively; inter- and intraassay coefficient of variation for all assays: <10%.

### Presentation of data and statistical analysis

Data in the table are given as means ± SEM and data in figures are demonstrated as box plots with the 5th, 25th, 50th (median), 75th, and 95th percentile. For comparison of medians, the Mann-Whitney test was used (SPSS/PC, v11.5; SPSS Inc, Chicago, IL, USA); *p *< 0.05 was the level of significance.

## Results

### Markers of inflammation in synovial tissue

In order to delineate severity of local tissue inflammation, we investigated lining layer thickness, overall cellularity, density of CD3+ T cells, CD163+ macrophages, and vascularity. Obviously, patients with RA had more severe inflammation as compared with patients with OA (Table 1).

### Conversion of DHEA into downstream steroid hormones in mixed synovial cells

**Figure 2:**
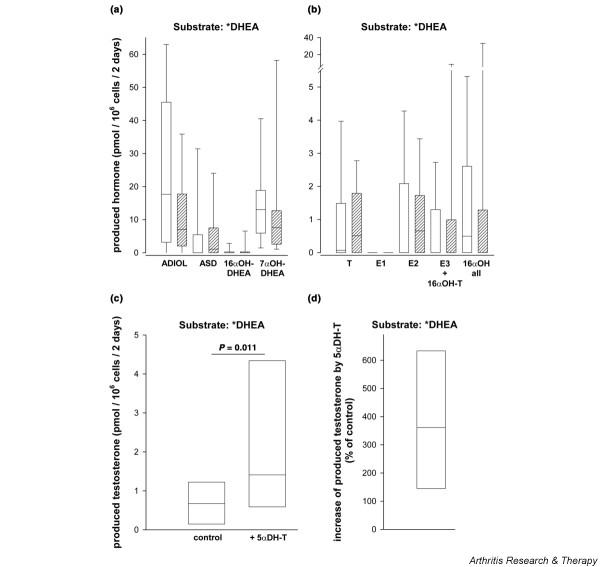
Conversion of DHEA into downstream steroid hormones in mixed synovial cells. **(a,b) **Spontaneous conversion of radiolabeled DHEA into downstream metabolites in OA (open bars, *n *= 24) and RA patients (hatched bars, *n *= 23). The E3 and 16αOH-testosterone produced are shown together in one bar because only one spot was detected for both steroids using TLC. The bar '16αOH all' reflects the sum of all 16α-hydroxylated products. **(c,d) **Conversion of radiolabeled DHEA into testosterone under the influence of 5α-dihydrotestosterone as investigated in three OA and one RA patient. All panels: values are given as pmol/10^6 ^cells/48 hr as box blots with the 5th, 25th, 50th (median), 75th, and 95th percentile when applicable. * Denotes radiolabeled substrate. OA, osteoarthritis; RA, rheumatoid arthritis. Other abbreviations are as given in the legend to Fig. 1.

DHEA is the major delta 5 androgen (Fig. 1), which is converted to androstenediol, ASD, 16αOH-DHEA, 7αOH-DHEA, testosterone, E1, E2, E3, and 16αOH-testosterone (Fig. 2a,b). The hormones produced did not differ between OA and RA (Fig. 2a,b). Interestingly, levels of testosterone were similar as compared with E2, combined E3 and 16αOH-testosterone (one spot in the chromatography), and the sum of all 16α-hydroxylated products (Fig. 2b). Neither gender nor therapeutic administration of non-steroidal anti-inflammatory drug (NSAIDs), leflunomide, or prednisolone influenced conversion of DHEA (data not shown).

Incubation of radiolabeled DHEA together with 5α-dihydrotestosterone demonstrated a marked increase of produced testosterone (Fig. 2c,d), which was not observed for androstenediol (mean: 90% of control without 5α-dihydrotestosterone; not shown in Fig. 2) and ASD (104%; not shown in Fig. 2). In addition, 5α-dihydrotestosterone tended to inhibit production of combined E3 and 16αOH-testosterone (63.9%, *p *= 0.068; not shown in Fig. 2).

### Conversion of ASD and testosterone into downstream steroid hormones in mixed synovial cells

**Figure 3:**
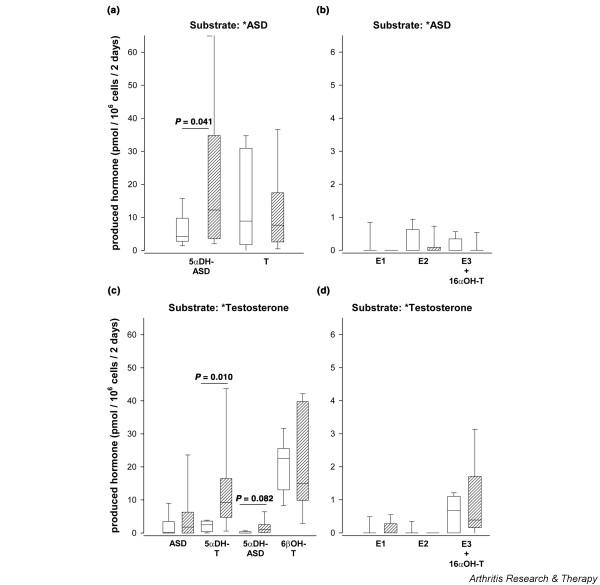
Conversion of ASD and testosterone into downstream steroid hormones in mixed synovial cells. **(a,b) **Spontaneous conversion of radiolabeled ASD into downstream metabolites in OA (open bars, *n *= 23) and RA patients (hatched bars, *n *= 19). **(c,d) **Spontaneous conversion of radiolabeled testosterone into downstream metabolites in OA (open bars, *n *= 10) and RA patients (hatched bars, *n *= 9). All panels: the E3 and 16αOH-testerone produced are given in one bar because only one spot was detected for both steroids using TLC. Values are given as pmol/10^6 ^cells/48 hr as box blots with the 5th, 25th, 50th (median), 75th, and 95th percentile when applicable. * Denotes radiolabeled substrate. OA, osteoarthritis; RA, rheumatoid arthritis. Other abbreviations are as given in the legend to Fig. 1.

ASD and testosterone are major delta 4 androgens (Fig. 1). Radiolabeled ASD was converted into 5α-dihydro-ASD, testosterone, and negligible amounts of E1, E2, E3, and 16αOH-testosterone (Fig. 3a,b). The level of 5α-dihydro-ASD produced was higher in RA as compared with OA (Fig. 3a). Radiolabeled testosterone was converted to ASD, 5α-dihydrotestosterone, 5α-dihydro-ASD, 6βOH-testosterone, and small quantities of E1 and E2 (Fig. 3c,d). Interestingly, using testosterone as the substrate led to small amounts of produced E3 and 16αOH-testosterone (one spot in the chromatography) (Fig. 3d). Similar to the results obtained with radiolabeled ASD, use of radiolabeled testosterone led to increased levels of 5α-dihydrotestosterone (*p *= 0.010, Fig. 3c) and 5α-dihydro-ASD (*p *= 0.082, Fig. 3c) in RA as compared with OA. Neither gender nor therapeutic administration of NSAIDs, leflunomide, or prednisolone influenced conversion of ASD and testosterone (data not shown).

### Aromatase expression in synovial tissue

**Figure 4:**
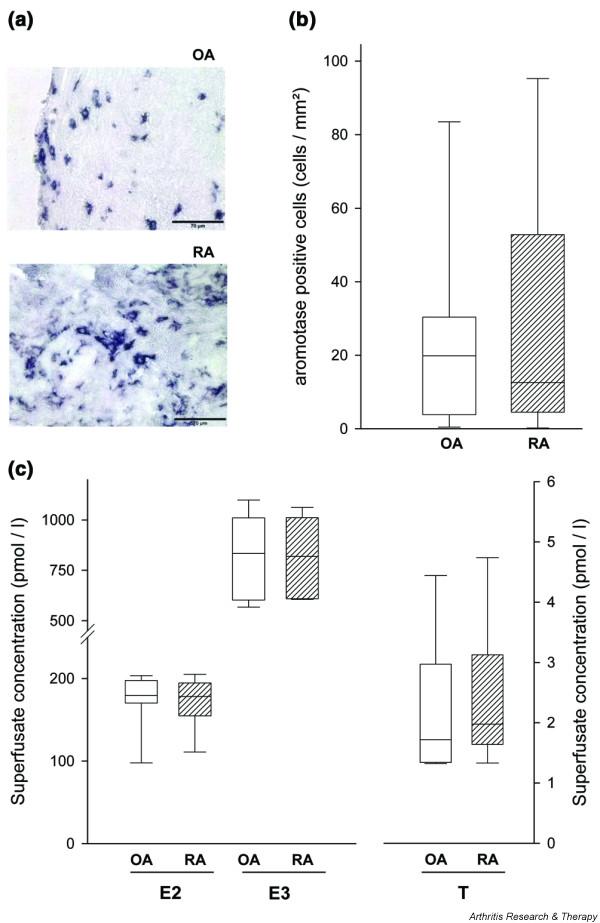
Aromatase expression in synovial tissue and endogenous steroid hormone release from superfused synovium. **(a) **Immunohistochemistry of aromatase in one OA and one RA patient. Using the respective control antibody revealed no staining of positive cells (not shown). Magnification: 400×. **(b) **Density of aromatase-positive cells in OA (open bars, *n *= 20) and RA patients (hatched bars, *n *= 16). **(c) **Spontaneously released E2, E3, and free testosterone from standardized superfused pieces of synovial tissue of OA (open bars, *n *= 20) and RA patients (hatched bars, *n *= 18). (b,c) Values are given as box blots with the 5th, 25th, 50th (median), 75th, and 95th percentile when applicable. OA, osteoarthritis; RA, rheumatoid arthritis. Other abbreviations are as given in the legend to Fig. 1.

Staining of synovial tissue in RA and OA patients demonstrated aromatase expression in the lining and sublining area in both patient groups (Fig. 4a). Quantitative analysis of aromatase expression in the tissue revealed that density of aromatase-positive cells was similar in RA and OA patients (Fig. 4b). Neither gender nor therapeutic administration of NSAIDs, leflunomide, or prednisolone influenced this result (data not shown).

### Endogenous steroid hormone release from superfused synovial tissue

In order to detect spontaneously released estrogens and testosterone, we superfused standardized synovial tissue slices and measured hormone concentrations in the superfusate. Hormone concentrations indicated the general presence of these hormones in the viable tissue in a quasi *in vivo *situation. Superfusate concentrations of E2, E3, and free testosterone were similar in RA and OA patients (Fig. 4c). It is obvious that concentrations of the two measured estrogens were increased in relation to free testosterone (Fig. 4c), which shows the preponderance of estrogens in relation to free testosterone. Neither gender nor therapeutic administration of NSAIDs, leflunomide, or prednisolone influenced superfusate concentrations of all three steroids (data not shown).

## Discussion

This study demonstrated three important new aspects of hormone conversion in primary synovial cells and synovial tissue of long-standing RA and OA patients in the advanced phase of the disease:

1. Conversion of DHEA yielded high amounts of estrogens and 16α-hydroxylated products in relation to testosterone (similar in RA and OA);

2. Conversion of ASD and testosterone particularly yielded androgens with elevated levels of 5α-hydroxylated androgens in RA as compared with OA (general blockade of aromatization and support of 5α-hydroxylation, particularly in RA);

3. Similarly in RA and OA, spontaneously released estrogens were markedly elevated in relation to free testosterone and aromatase expression was similar in the two disease groups. All effects were independent of gender and therapeutic administration of NSAIDs, leflunomide, or prednisolone.

Delta 4 androgens such as testosterone and ASD inhibit secretion of IL-1β, IL-6, TNF, and other proinflammatory mediators [1-7]. The more potent, pure androgen 5α-dihydrotestosterone inhibits the NFκB-mediated activation of the human IL-6 gene promoter in human fibroblasts and T cell proliferation in animal models [8,9]. From this information and from our present study, it is very likely that therapy with ASD and testosterone can be beneficial in RA patients. Indeed, two therapeutic studies with testosterone have demonstrated remarkable benefits in male and female patients with RA [10,11]. This is quite different when using DHEA because, as shown here, DHEA is converted to proproliferative 16α-hydroxylated estrogens. Indeed, one open-label study in RA patients with DHEA demonstrated no beneficial effects [23]. Our study confirms that administration of DHEA is most probably not a favorable therapy in RA whereas ASD and testosterone might be used to treat RA patients.

At this point the question arises as to how ASD and testosterone can inhibit aromatization of androgens in synovial cells. Normally, one would expect that administered androgens are rapidly aromatized, thus increasing the amounts of downstream estrogens [13,24]. Furthermore, androgens can also increase aromatase gene expression [24]. As demonstrated here for the first time, this seems to be largely different in synovial cells of patients with RA and OA because ASD and testosterone suppress aromatization. Interestingly, in cultured human skin fibroblasts, incubation with ASD or testosterone resulted in a similar decline in aromatase activity [25]. This is further supported by a study with granulosa cells, which demonstrated that ASD and testosterone are able to inhibit aromatase activity as well [26]. The reasons for stimulation or inhibition of the aromatase in different cells types under different conditions are not yet known. In addition, we do not know whether administered androgens inhibit the enzyme directly (substrate inhibition) or inhibit aromatase gene expression (genomic action) in synovial cells of OA and RA patients. This mechanism of action requires further study.

Other important findings in this study of OA and RA patients are the similar aromatase expression and identical concentrations of produced and released synovial estrogens, irrespective of therapy and gender. We recently demonstrated that estrogen synovial fluid levels were higher in RA as compared with traumatic controls, irrespective of gender [17]. Thus, it seems that OA patients are largely different from traumatic knee joint patients. This underlines that inflammation in OA, apparently similar to that in RA, up-regulates aromatase activity leading to elevated levels of estrogens in synovial tissue. We have to emphasize again that OA and RA patients suffer from similar chronic inflammatory diseases in the advanced phase, best demonstrated by similar vascularity. In this phase of the disease, neoangiogenesis is most probably not an important aspect of the disease. Since serum estrogen levels are increased in RA patients as compared with OA patients or healthy controls, serum estrogens in RA might be released from another source such as fat tissue. Since synovial estrogen production is not different in the two diseases, up-regulation of aromatization in fat tissue in RA patients would explain higher serum and synovial fluid levels of estrogens in RA compared with OA or healthy controls. These findings support the concept of a systemic inflammatory disease in RA involving other hormone conversion sites, which is largely different in OA. In addition, this present study demonstrated that concentrations of produced (when using DHEA) and released estrogens are high in relation to androgens. This corroborates the findings in RA synovial fluid where estrogens were also higher in relation to androgens [17]. This generally supports the concept of increased aromatization in synovial tissue under inflammatory conditions.

A further important finding in this study were the relatively high quantities of 16α-hydroxylated estrogens in synovial cell culture experiments (when using DHEA) and superfusion experiments (looking at E3), which was irrespective of therapy and gender. Typically serum levels of E3 in non-pregnant women and men are below 7,000 pmol/l [27]. This can increase during pregnancy up to 100,000 pmol/l. Serum levels of free testosterone are approximately 35 pmol/l (women) and 350 pmol/l (men) [27]. In the present study, we used a superfusion flow rate of 66 μl/min, which reflects the tissue perfusion rate found in the interstitial space. Under these conditions, superfusate E3 concentration in RA and OA patients reached a level of approximately 750 pmol/l whereas levels of free testosterone were approximately 2 pmol/l. Thus, the relationship of E3 to testosterone was 375:1 in the synovial tissue superfusate of RA and OA patients whereas it is typically 20 (men) and 200 (women):1 in the serum. Under additional consideration of other 16α-hydroxylated products (16αOH-testosterone), this obviously demonstrates that generation of 16α-hydroxylated products is markedly increased in relation to testosterone in synovial tissue of RA and OA patients. Studies in breast cancer research delineated that 16α-hydroxyestrogens are mitogenic and proproliferative [28-30]. In proliferation assays, 16α-hydroxyestrogens had an activity comparable with that observed for the carcinogen 7,12-dimethylbenz [a]anthracene (i.e. DMBA)[30]. Thus, 16α-hydroxyestrogens may induce a hyperestrogenic proinflammatory state. This is particularly true if the naturally occurring anti-estrogens, the 2-hydroxylated estrogens, are diminished, which has recently been demonstrated [31]. In our present study, we did not detect even minimal amounts of 2-hydroxylated estrogens, which clearly supports the obvious preponderance of 16α-over 2-hydroxylated estrogens.

It is interesting that all observed conversion results did not differ between male and female patients. One might expect that androgen conversion to estrogens is increased in female as compared with male patients. However, on the local level of macrophage-mediated androgen conversion, no obvious differences exist between the gender groups. It is well-known that female patients have an increased incidence of autoimmune diseases. Thus, it seems obvious that circulating estrogens from the ovaries support the autoimmune process in the reproductive phase of a woman. This is most probably due to the estrogenic support of the adaptive immune system [32,33]. In RA patients, this might well happen 10 years before disease outbreak because autoimmune phenomena are present in the presymptomatic phase of the disease [34]. However, in the advanced phase of the destructive joint disease, when other cell types such as macrophages, neutrophils, and fibroblasts play a local inflammatory role, circulating estrogens are less important (postmenopausal). In this latter situation, estrogens are locally converted from circulating adrenal prehormones such as DHEAS and androstenedione, the serum levels of which are not largely different between male and female subjects. This aspect and the fact that macrophages as well as fat cells convert prehormones independently of gender, explain the similar results in female and male patients.

At this point, the next important question appears to be whether, or not, these changes are specific for RA patients. We think that observed differences between OA and RA patients (5α-hydroxylation) are not specific for RA patients because, most probably, increased hormone conversion has not been evolutionarily conserved for a specific disease. We recently demonstrated a concept regarding why most of the observed changes during the symptomatic phase of an inflammatory disease, particularly in the symptomatic phase, are not specific for a certain inflammatory process [35]. This theory, presentation of which goes beyond the scope of this article, can explain why many similar phenomena appear in very different chronic inflammatory diseases [35].

## Conclusion

This study revealed that synovial tissue of patients with RA and OA demonstrated increased aromatization and 16α-hydroxylation irrespective of gender and therapy. These stimulated, central enzyme pathways can be inhibited by administration of the two androgens ASD and testosterone. This study provides a further rationale to treat RA patients with ASD and testosterone in order to inhibit aromatization and 16α-hydroxylation and to increase availability of local androgens. Further studies are needed to investigate the molecular mechanisms as to how ASD and testosterone are able to inhibit these two important proinflammatory enzyme pathways in synovial cells of RA and OA patients.

## Abbreviations

APAAP = alkaline phosphatase-anti-alkaline phosphatase; ASD = androstenedione; CD = cluster of differentiation; DHEA = dehydroepiandrosterone; DMBA, 7,12-dimethylbenz[a]anthracene; E1 = estrone; E2 = 17β-estradiol; E3 = estriol; FCS, fetal calf serum; HE = hematoxylin-eosin; NSAID = non-steroidal anti-inflammatory drug; OA = osteoarthritis; OH = hydroxy- or hydroxylated; PBS = phosphate buffered saline; RA = rheumatoid arthritis; RP-HPLC = reverse-phase high-performance liquid chromatography; RPMI medium = Rose Park Memorial Institute medium; TLC = thin-layer chromatography.

## Competing interests

The author(s) declare that they have no competing interests.

## Authors' contributions

MS participated in the concept and design, acquisition, interpretation and analysis of data. CW and HN participated in acquisition and analysis of data. SA dealt with acquisition of data and revision of the article. JS participated in drafting and revising the article. RHS participated in the concept and design, analysis and interpretation of data, and drafting and revising the article.

## Figures and Tables

**Table 1:** Characteristics of patients under study

	Osteoarthritis	Rheumatoid arthritis
*n*	26	25
Age, years	71 ± 2 [55–88]	66 ± 2 [39–84]
Gender, F/M	21/5 (81/19)	19/6 (76/24)
Disease duration, years^§^	5 ± 1	10 ± 1
Erythrocyte sedimentation rate, mm/1st hr	16.6 ± 3.2	35.2 ± 7.2**
C-reactive protein, mg/l	6.6 ± 0.9	26.3 ± 7.5^#^
Rheumatoid factor positive	1/26 (0.5)	12/25 (48.0)
Lining layer, cells	2.1 ± 0.1	3.9 ± 0.3^##^
Cell density, cells/mm^2^	1257 ± 121	1864 ± 202**
T cell density, cells/mm^2^	22.2 ± 4.7	58.8 ± 20.1
CD163^+ ^macrophage density, cells/mm^2^	54.7 ± 10.1	101.3 ± 20.5*
Vascularity, vessels/mm^2^	28.8 ± 2.9	31.1 ± 3.7
Medication		
Prednisolone	n.a.	19 (76.0)
Mean prednisolone dose, mg/d	n.a.	5.2 ± 0.9
NSAIDs	14/26 (54.0)	20/25 (80.0)
Methotrexate	n.a.	4/25 (16.0)
Leflunomide	n.a.	8/25 (32.0)
Azathioprine	n.a.	1/25 (4.0)
Sulfasalazine	n.a.	1/25 (4.0)
Hydroxychloroquine	n.a.	4/25 (16.0)
Cyclosporin A	n.a.	1/25 (4.0)
Anti-TNF therapy strategies	n.a.	0/25 (0.0)

*p = 0.065, **p < 0.05, ^#^*p *< 0.01, ^##^*p *< 0.001 for the comparison versus osteoarthritic patients. ^§^Disease duration in OA patients is a rough estimate because the exact starting point is often not precisely known. Data are given as means ± SEM, percentages in parentheses, and ranges in square brackets. F/M, female/male; n.a., not applicable; NSAIDs, non-steroidal anti-inflammatory drugs; TNF, tumor necrosis factor.
